# Exposure to occupational risk factors is associated with the severity and progression of chronic obstructive pulmonary disease

**DOI:** 10.1097/MD.0000000000032908

**Published:** 2023-02-10

**Authors:** Yao Chen, Cong Peng, Hua Zhang, Yu Cai, Rui Yuan, Pingping Song, Chunling Zhang, Yongjian Yan

**Affiliations:** a School of Public Health, Qingdao University, Qingdao, Shandong Province, China; b Shandong Academy of Occupational Health and Occupational Medicine, Shandong First Medical University & Shandong Academy of Medical Sciences, Jinan, Shandong Province, China; c Health Bureau of Daiyue District, Tai’an, Shandong Province, China; d The Affiliated Qingdao Central Hospital of Qingdao University, The Second Affiliated Hospital of Medical College of Qingdao University, Qingdao, Shandong Province, China; e Shandong First Medical University & Shandong Provincial Hospital, Jinan, Shandong Province, China.

**Keywords:** chronic obstructive pulmonary disease (COPD), computerized medical record, occupational exposure, retrospective study, risk factors, work-related disease

## Abstract

Chronic obstructive pulmonary disease (COPD) results from a complex interaction between genes and the environment, and occupational exposures are an underappreciated risk factor. Until now, little research attention has been paid to the potential impact of occupational risk factor exposure on the COPD in China. The aim of this retrospective study was to analyze the role of occupational risk factor exposure on the severity and progression of COPD for exploring new prevention strategies for this disease. This study adopted a random cluster-sampling method. Five grade-A tertiary hospitals that met the inclusion criteria were selected as the survey sites, and patients with COPD hospitalized in these hospitals from January 1, 2019, to December 31, 2019, were selected as the research subjects. Data of the patients diagnosed with COPD met the Global Initiative for Chronic Obstructive Lung Disease (2019) criteria and were collected from the computerized medical record databases. Among 4082 investigated COPD patients, 1063 (26%) were found to have occupational risk factor exposure history. The top 3 industries with a large COPD case number and a history of occupational risk factor exposure ranked in the order of agriculture (including farming, forestry, animal husbandry, and fishery), manufacturing, and mining. Further multivariate logistic regression analysis indicated that when setting a low exposure level as a reference, medium and high exposure levels were correlated with the severity of COPD (odds ratio values were 2.837 and 6.201, respectively, *P* < .05). Linear regression analysis showed that cumulative exposure to occupational risk factors was negatively correlated with the forced expiratory volume in 1-second percentage of COPD patients, with a correlation coefficient of 0.68. Our results indicated that occupational risk factor exposure levels were related to the severity of COPD significantly. The incubation period of COPD in the exposure group was significantly shorter than that in the non-exposure group. To prevent worked-related COPD, special attention and control efforts should be taken to reduce the level of occupational risk factors such as organic dust, irritating chemicals, etc in the work environments, especially in the industries of agriculture, forestry, animal husbandry and fishery, manufacturing, and mining.

## 1. Introduction

Chronic obstructive pulmonary disease (COPD), a common and preventable disease, is characterized by persistent respiratory symptoms and airflow limitation caused by abnormal airways and alveoli resulting from massive exposure to toxic particles or gases.^[1]^ In 2018, a large-scale population study demonstrated that the number of adult COPD patients in China had increased by 67% in the past 10 years.^[2]^ Epidemiological and experimental research data have demonstrated that the risk factors for COPD include host factors, respiratory infections, tobacco, occupational hazard factors, and environmental exposures.^[3]^ Among these factors, occupational hazard exposure is the least studied factor of COPD. Studies have estimated that 20% of COPD cases might be attributable to occupational hazard exposure, and the population-attributable ratio of the contribution of occupational factors to COPD burden is between 15% and 20%.^[4]^ However, occupational factors have been largely ignored at the onset of COPD in clinical practice. According to current occupational disease data, China has reported <100 cases of worked-related COPD annually^[5]^ which is far lower than the estimated number. If patients suffering from work-related COPD cannot be diagnosed in time, this disease will be repeatedly aggravated by re-exposure to the occupational risk factors. Therefore, more attention should be paid to the role of exposure to occupational risk factors in the onset of COPD.

The aims of this study included: identifying the industries as well as the occupational risk factors related to the onset of COPD; provide reference information for early prevention, diagnosis, and treatment of worked-related COPD.

## 2. Methods

### 2.1. Subjects

According to the administrative division of Shandong Province (East, West, Middle, South, and North regions), one city was randomly selected from each region. Five cities in total including Qingdao, Liaocheng, Jinan, Zaozhuang, and Weifang were randomly sampled. One grade-A tertiary hospital was randomly selected from each city as the survey site. COPD patients hospitalized in these hospitals from January 1, 2019, to December 31, 2019, were investigated as the study subjects. The inclusion criteria included: age ≥ 18 years old, clear diagnosis based on the Global Initiative for Chronic Obstructive Lung Disease (GOLD) criteria 2019, and clinical information and data registration. The exclusion criteria included: severe cardiovascular and cerebrovascular diseases; chest and abdominal surgery in the past 2 months; other lung airflow limitation diseases such as acute respiratory infection, bronchiectasis, tuberculosis, and lung cancer; mental illness or cognitive impairment; and subjects who were unable to communicate or not willing to participate in this study.

This study was reviewed and approved by the Medical Ethics Committee of the Shandong Institute of Occupational Health and Occupational Disease Prevention, and informed consent was obtained from all participants.

### 2.2. Methods

Information on the selected COPD patients was downloaded from the computerized medical record database from each hospital. The contact information included in the medical records and a self-designed questionnaire named “Onset of COPD patients and exposure to occupational risk factors aggravating disease” were used for the telephone follow-up survey (Supplementary Table S1, Supplemental Digital Content, http://links.lww.com/MD/I445). Before the research started, all investigators who participated in this study had received uniform investigation processes training.

### 2.3. Diagnostic criteria and index definitions

#### 2.3.1. Diagnostic criteria.

According to GOLD guidelines (2019), patients with dyspnea, cough, expectoration, repeated lower respiratory tract infections, and exposure to risk factors with a forced expiratory volume in 1 second (FEV_1_)/forced vital capacity (FVC) < 0.7 (after administering bronchodilators) and excluding other known lung diseases were diagnosed as COPD.

#### 2.3.2. Indicator definition.

Quantification of cigarette smoking: the pack year was calculated by multiplying the number of packs of cigarettes smoked per day by the number of years an individual had smoked.

Occupational risk factor exposure refers to exposure to occupational risk factors in one’s current work and/or previous work (including farm work), where the worker worked for at least 8 hours a day, 5 days a week, and the cumulative exposure time exceeded 1 year.^[6]^

Types of occupational risk factors: the International Labor Organization classifies occupational exposure risk factors that cause COPD as “vapor, gas, dust, smoke, or their compound.” At present, occupational risk factors that cause COPD are generally divided into 2 categories: irritant compounds and dust. The dust is divided into inorganic dust (mineral dust, carbon-containing dust, metallic dust, artificial inorganic dust), organic dust (animal dust, plant dust, artificial organic dust), and mixed dust. All irritating vapors, gases, and fumes are collectively referred to as irritating chemicals, including acids, alkalis, halogens, compounds, aldehydes, esters, ethers, epoxy compounds, and other volatile gases and combustion fumes.^[7]^

Occupational risk factor exposure level assessment: since the follow-up subjects could not provide accurate information on the exposure concentrations, occupational exposure in the workplace was divided into 3 levels based on the working environment and personal protection described by the patient. Low exposure level applied to workplaces with advanced technology, high degree of automation, mechanization, airtightness, clean working environment, and better protective measures. Medium exposure level applied to workplaces with average technological advancement, average automation, mechanization, airtightness, and environmental and personal protection, but the protection measures could not achieve the expected results. High exposure level applied to workplaces with backward technology, low level of automation/mechanization, manual or non-enclosed operation environment, no environmental protection or ineffective protection, and no personal protection or ineffective protection. These three types of exposure levels in the work environment were respectively assigned values of 1, 2, and 3. The cumulative exposure level was calculated as the exposure level multiplied by the actual number of exposure years to the occupational risk factors.^[8]^

### 2.4. Statistical analysis

After the questionnaire follow-up, the number of questionnaires was counted, and the quality of the questionnaires was reviewed. A total of 628 invalid questionnaires (those with a blank rate ≥ 10%) were eliminated. EpiData 3.1 (The EpiData Association) was used to input the valid questionnaire data and export it to the Excel database (Microsoft). Statistical analysis was performed using SPSS 23.0 software (IBM Corporation). For count data, relative number indicators (rate, composition ratio, and relative ratio) were used to describe the distribution. For measurement data, the mean ± standard deviation was used to describe those with a normal distribution; and the median and interquartile range (P25–P75) were used to describe those with a non-normal distribution. Enumeration data were compared between groups using the chi-square test or Fisher exact probability test. Multiple logistic regression was used for multivariate analysis. A bivariate correlation analysis of data with a normal distribution was performed using a linear regression model. The inspection level was set to 2-sided *α* = 0.05, and *P* < .05 was considered statistically significant.

## 3. Results

### 3.1. Analysis of the basic characteristics of the subjects

A total of 4710 subjects were included according to the inclusion and exclusion criteria. Questionnaires that did not meet the criteria were excluded. A total of 4082 valid questionnaires were obtained, with an effective rate of 86.7%.

Depending on whether they were exposed to occupational risk factors, subjects were divided into an exposure group and a non-exposure group. There were 1063 (26.0%) patients in the exposure group and 3019 (74.0%) in the non-exposure group. The differences between the 2 groups in terms of age of onset, gender, education level, and smoking history were shown in Table 1.

**Table 1:** Comparison of the basic characteristics between occupational risk factor exposure and non-exposure groups.

Index	Variable	Case Number (n)	Exposure cases (%)	Non-exposure cases (%)	χ^2^/*t* value	*P* value
Age of onset (yr old)	30 ~	18	18 (1.7)	0 (0.0)	824.17	<.001
	40 ~	53	48 (4.5)	5 (0.2)		
	50 ~	310	281 (26.5)	29 (0.9)		
	60 ~	1009	384 (36.1)	625 (20.7)		
	70 ~	1603	272(25.6)	1331 (44.1)		
	80 ~	1089	60 (5.6)	1029 (34.1)		
Gender	Male	2679	622 (58.5)	2057 (68.1)	32.26	<.001
	Female	1403	441 (41.5)	962 (31.9)		
Education level	Elementary school and below	2475	930 (87.5)	1545 (51.2)	434.98	<.001
	Middle school	1390	120 (11.3)	1270 (42.1)		
	College degree and above	217	13 (1.2)	204 (6.7)		
Smoking history	Current smoker	1344	284 (26.7)	1060 (35.1)	45.31	<.001
	Former smoker	1373	339 (31.9)	1034 (34.3)		
	Never smoker	1365	440 (41.4)	925 (30.6)		

#### 3.1.1. Analysis of the age of onset.

**Figure 1.**
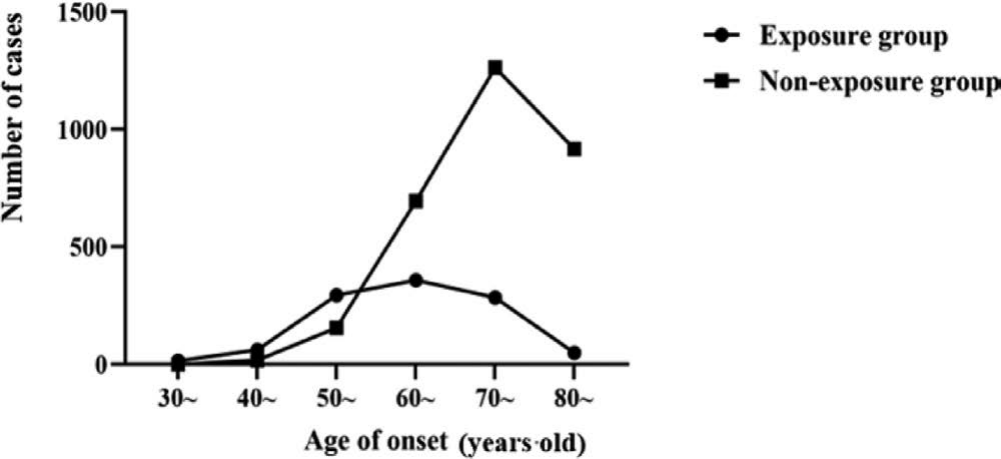
Distribution of age of onset between occupational risk factor exposure and non-exposure groups.

The age of onset distribution between the exposure and non-exposure groups (65.31 ± 10.13 and 74.93 ± 8.19 years old, respectively) was significantly different (*P* < .01). When analyzed by age group, the number of patients in each age group was significantly different between the 2 groups (*P* < .01). Among them, almost all patients under 40 years old had a history of occupational risk factor exposure, and 347 cases (91.1%) developed COPD under 60 years old (Fig. 1).

#### 3.1.2. Gender distribution.

**Figure 2.**
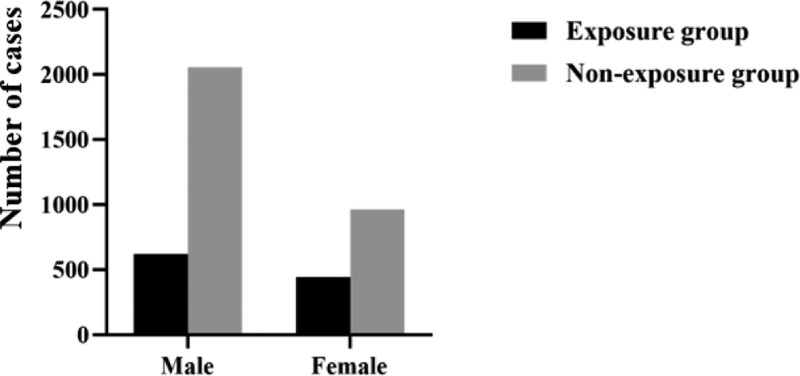
Gender distribution between occupational risk factor exposure and non-exposure groups.

Among 4082 patients, 2679 (65.6%) were males and 1403 (34.4%) were females. In the exposure group, there were 622 males (58.5%) and 441 females (41.5%). The difference in gender distribution between the 2 groups was statistically significant (*P* < .05) (Fig. 2).

#### 3.1.3. Analysis of education level.

**Figure 3.**
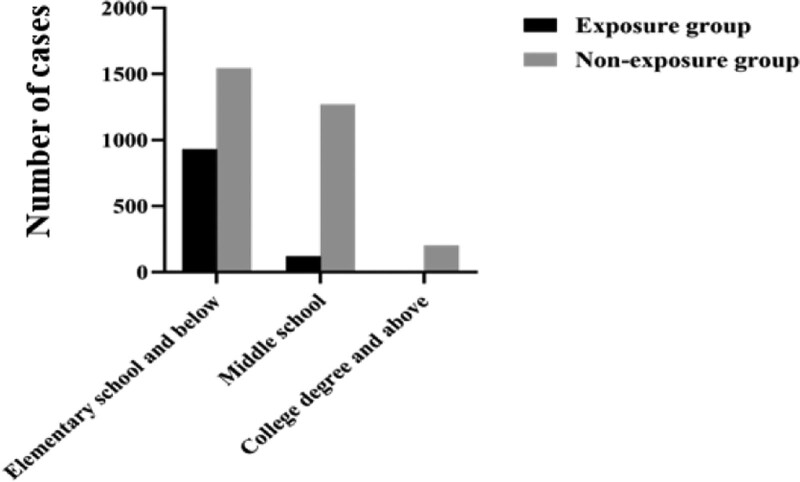
Distribution of education level between occupational risk factor exposure and non-exposure groups.

The difference in the distribution of education level between 2 groups was statistically significant (*P* < .05). A higher occupational risk factor exposure rate was found in the lower education level cases, reflecting a lack of knowledge or awareness of workplace self-protection in the lower education level populations (Fig. 3).

#### 3.1.4. Analysis of smoking status.

Among 4082 investigated COPD patients, 2717 cases (66.6%) were smokers, and of these, 2094 (69.4%) cases were in the non-exposure group. The difference in the distribution of smoking history between the 2 groups was statistically significant (*P* < .05). There were 623 patients with both occupational exposure and smoking history, accounting for 58.6% of the exposed persons and 15.3% of the total number of subjects.

#### 3.1. 5. The incubation period of COPD.

The incubation period of COPD in the exposure group was significantly shorter than that in the non-exposure group (*P* < .05) (Table 2).

**Table 2:** Comparison of the incubation period between occupational risk factor exposure and non-exposure groups.

Index	Exposure cases (yr)	Non-exposure cases (yr)	*t* value	*P* value
Disease incubation period	11.02 ± 5.32	15.68 ± 6.35	15.08	.000

### 3.2. Exposure to occupational risk factors in COPD patients

Among 4082 investigated COPD patients, 1063 (26.0%) were exposed to occupational risk factors. Types of exposure, working age, exposure level, industry categories, work environment, as well as personal protection etc were analyzed.

#### 3.2.1. Distribution of occupational risk factors.

##### 3.2.1.1. Dust.

Among the 1063 patients with occupational risk factor exposure history, 841 (79.1%) were exposed to dust. Among them 684 cases (81.3%) were exposed to organic dust, 126 cases were exposed to inorganic dust (15.0%), and 31 cases were exposed to mixed dust (3.7%). Patients exposed to organic dust plus plant dust were 651 cases (95.2%), and 33 cases (4.8%) were exposed to animal dust. Patients exposed to inorganic dust plus carbon-containing dust were 75 cases (59.5%); 32 cases (25.4%) patients were exposed to artificial inorganic dust, 12 cases (9.5%) were exposed to mineral dust, and 7 cases (5.6%) exposed to metallic dust.

##### 3.2.1.2. Irritating chemicals.

Among the 1063 patients, 222 cases (20.9%) were exposed to irritating chemicals. Seventy-one cases (48.3%) were exposed to acids and acid-forming compounds, and 24 cases (16.3%) were exposed to aldehydes. The exposure rates to hydrochloric acid, sulfuric acid, and sulfur dioxide for acid and acid-forming compounds were 34.7%, 33.3%, and 27.8%, respectively. The types of occupational risk factors identified in this study are shown in Table 3.

**Table 3:** Types of occupational risk factors.

Types	Subtypes	Classification
Dust	Organic dust	Plant dust (grain dust, cotton dust, wood dust, paper dust, etc.). Animal dust (mixed animal dust, fur, etc.)
	Inorganic dust	Contains carbon dust (coal dust, carbon black, etc.)
		Artificial inorganic dust (cement, artificial mineral wool, artificial inorganic mixed dust, etc.)
		Mineral dust (silica dust, asbestos, talc, etc.)
		Metallic dust (metal mixed dust, iron, aluminum, copper, lead, etc.)
	Mixed dust	N/A
Irritating chemicals	Acids and acid-forming compounds	Hydrochloric acid, sulfur dioxide, sulfuric acid, nitric acid, hydrogen fluoride, etc
	Aldehydes	Formaldehyde, acetaldehyde, etc
	Nitrogen oxides	Nitric oxide, nitrogen dioxide, etc
	Metal compound smog	Copper oxide, iron oxide, manganese dioxide, copper hydroxide, iron hydroxide, etc
	Organic solvents	Benzene, toluene, xylene, vinyl chloride pyrethroid, etc.
	Chlorine and its compounds	Chlorine, hydrogen chloride, etc
	Ammonia	Ammonia, etc.

#### 3.2.2. Industry distribution.

**Figure 4.**
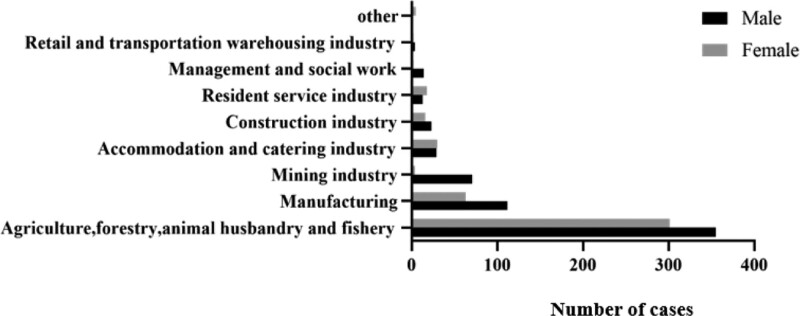
Distribution of occupational risk factors among different industries (n = 1063).

Top 3 industries with a large number of COPD cases are agriculture (including farming, forestry, animal husbandry, and fishery), manufacturing, and mining (Fig. 4).

The major occupational risk factors exposed in different industries were significantly different. Patients who worked in agriculture, forestry, animal husbandry, and fishery industries were mainly exposed to dust (grain dust 92.4% and animal mixed dust 5.0%). While those who worked in the manufacturing industries were exposed to acids and/or acid-forming compounds (44.6%) and inorganic dust (19.4%). In the mining industry, patients were mainly exposed to coal dust (81.3%) and silicon dust (8.0%).

#### 3.2.3. Investigation of major work types in the top 3 industries with a large number of COPD cases.

Major types of work in the top 3 industries with a large number of COPD are shown in Table 4.

**Table 4:** Major types of work in the top 3 industries with a large number of COPD cases.

Industry categories	Major types of work
Agriculture (including farming, forestry, animal husbandry and fishery)	Grain planting, pasture processing, lawn planting, livestock raising
Mining	Coal mining, coal washing, oil mining, ore mining
Manufacturing	Metal smelting, metal tool manufacturing, grain milling, textile processing (cotton, wool, hemp, silk, leather), wood processing, paper processing, petroleum and coal fuel processing, chemical product manufacturing, printing, and dyeing

COPD = chronic obstructive pulmonary disease.

#### 3.2.4. Distribution of exposure levels.

According to exposure intensity to the occupational risk factors, patients were divided into 3 types: low, medium, or high exposure levels. The numbers of patients in the 3 groups were 119, 328, and 616 respectively. Among them, the number of patients with high exposure levels was the biggest accounted 57.9%.

### 3.3. Association between occupational risk factor exposure and COPD

According to the severity of airflow limitation, 1063 patients with a history of exposure to occupational risk factors were divided into mild and severe groups. The effects of exposure duration, exposure level, and exposure types of occupational risk factors on patients’ conditions were analyzed. The results showed only the exposure levels were significantly related to the severity of COPD (*P* < .05) (Table 5).

**Table 5:** Univariate analysis of occupational risk factor exposure and COPD.

Index	Variable	Severity of airflow imitation		χ^2^ value	*P* value
		Mild	Severe		
Exposure duration (yr)	<10	122 (22.5%)	99 (19.0%)	2.58	.462
	10 ~	101 (18.6%)	91 (17.5%)		
	20 ~	86 (15.8%)	88 (16.9%)		
	≥30	234 (43.1%)	242 (46.6%)		
Exposure level (value)	Low exposure	96 (17.7%)	23 (4.4%)	68.17	.000
	Medium exposure	189 (34.8%)	139 (26.7%)		
	High exposure	258 (47.5%)	358 (68.9%)		
Exposure types	Dust	435 (80.1%)	406 (78.1%)	0.665	.415
	Irritating chemicals	108 (19.9%)	114 (21.9%)		

COPD = chronic obstructive pulmonary disease.

#### 3.3.1. Multiple logistic regression analysis on occupational risk factor exposure and COPD.

The severity of COPD was set as the dependent variable, the exposure level (X1) was set as the independent variable and the smoking status and age were set as covariates to perform multiple logistic regression analysis to further screen risk factors. After adjusting for confounding factors, the results showed that exposure to occupational hazard factors was a risk factor that affected the severity of COPD patients. When considering the low exposure level as a reference, the medium and high exposure levels were correlated with the severity degree of work-related COPD. The odds ratio (OR) and 95% confidence interval (CI) values were 2.837 (1.702, 4.729) and 6.201 (3.801, 10.118), respectively (*P* < .05 Table 6).

**Table 6:** Multivariate analysis of occupational risk factor exposure and COPD.

Index	Variable	*B*	SE	Wald	*P*	OR	95% CI for Exp(B)	
							Lower	Upper
Age of onset		−0.17	0.007	6.648	.01	0.983	0.970	0.996
Smoking history		0.220	0.096	5.242	.022	1.246	1.032	1.503
Exposure level	Low exposure Medium exposure High exposure	1.00 (ref.)						
		1.043	0.261	16.013	.000	2.837	1.702	4.729
		1.825	0.250	53.368	.000	6.201	3.801	10.118

The Mild COPD group was used as the reference group. The medium and high exposure levels were correlated with the severity degree of work-related COPD, the odds ratio (OR) and 95% confidence interval (CI) values were 2.837 (1.702, 4.729) and 6.201 (3.801, 10.118), respectively (*P* < .05).

CI = confidence interval, COPD = chronic obstructive pulmonary disease, OR = odds ratio.

#### 3.3.2. The impact of cumulative exposure to occupational risk factors on the lung function of COPD patients.

**Figure 5.**
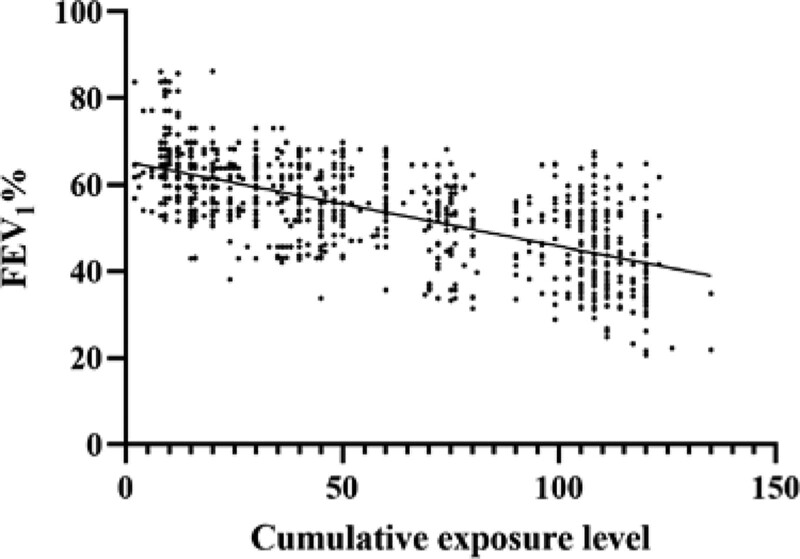
Occupational exposure levels in the workplace was divided into 3 types: low, medium, and high exposure levels. These 3 types were assigned values of 1, 2, and 3, respectively. The cumulative exposure level was calculated as the exposure level multiplied by the actual number of exposure years to the occupational risk factors.

The cumulative exposure to occupational risk factors was set as the independent variable and the FEV_1_% of COPD patients was set as the dependent variable for linear regression analysis. The results showed that cumulative exposure to occupational risk factors was linearly negatively correlated with FEV_1_%. The more cumulative exposure, the worse the lung function of COPD patients became. The correlation coefficient *r* was 0.68, the linear regression equation was *Y* = −0.1955 *X* + 65.39, and the regression equation was statistically significant (*P* < .05) (Fig. 5).

## 4. Discussion

### 4.1. Occupational risk factor exposure of COPD patients

According to American Thoracic Association, occupational risk factor exposure accounts for 10 to 20% of the COPD cases.^[9]^ In this study, among 4082 COPD investigated patients, 1063 cases were found to have a history of exposure to different kinds of occupational risk factors during their occupational career (accounted for 26.0%) which is higher than that American Thoracic Association reported. Females in the exposure group accounted for 41.5%, but 30.2% in the non-exposure group, suggesting that occupational risk factor exposure may have a greater impact on females than males.

In a study of 94,551 never-smokers in the population-based UK Biobank cohort, Matteis^[10]^ found that manufacturing is the sector with the highest risk of COPD, and agriculture also has a significantly increased risk of COPD. Hnizdo^[11]^ conducted a study on 9823 US population-based Third National Health and Nutrition Examination Survey and the results showed that ORs for COPD were increased for the following industries: rubber, plastics, and leather manufacturing; utilities; office building services; textile mill products manufacturing; the armed forces; food products manufacturing; repair services and gas stations; agriculture; sales; construction; transportation and trucking; personal services; and health care. Our study found that the occupational risk factor exposure rates among the COPD patients in different industries were different. The top 3 industries with a large number of COPD cases and a history of exposure to occupational risk factors were agriculture (including farming, forestry, animal husbandry, and fishery), manufacturing, and mining. There are many occupational hazards in these types of work environments. For example, people engaged in agriculture can be exposed to grain dust; those engaged in animal husbandry can be exposed to animal dust; those engaged in coal mining can be exposed to coal dust, silica dust, or metallic dust; and those engaged in chemical manufacturing can be exposed to irritating gases and fumes. Workers engaged in these industries and occupations normally were not very well educated resulting in insufficient knowledge of occupational risk factor exposure and low awareness of personal protection. Therefore, special attention should be paid to the prevalence, prevention, and control of COPD in these industries and occupations.

A British study reported that occupational exposure to dust occurs in many industries, such as coal mining, gold mining, agricultural production, and high-content dust operations in the cotton textile industry.^[12]^ In 2000, >20 million people in China were registered as being exposed to dust. There were also many unregistered contract workers and shift workers who were exposed to dust. In addition, China is the world’s largest agricultural producer by volume, and the dust exposure rate during farmland production operations is relatively high. This study showed that the number of patients with a history of exposure to occupational risk factors with COPD accounted for 79%. Among the occupational dust risk factors, the exposure rate of organic dust such as grain dust was the highest accounting for 95.2%. From 2010, the International Labor Organization started to report the global number of COPD caused by inhalation of coal dust, dust from stone quarries, wood dust, dust from cereals and agricultural work, dust in animal stables, dust from textiles, and paper dust, arising from work activities as an occupational disease.^[7]^ Therefore, China should develop “Standards for the Diagnosis of Occupational Dust-induced COPD” as soon as possible.

### 4.2. Association between exposure to occupational risk factors and COPD

Studies have demonstrated that COPD is related to the level of exposure to occupational risk factors. Pualin^[13]^ found that people with job exposures had higher odds of COPD (OR, 1.44; 95% CI, 1.04–1.97). Marchetti^[14]^ investigated 9614 African Americans or non-Hispanic whites and found that occupational exposure to both dust and fumes was reported by 47.9% of men and 20.1% of women, Adjusting for age, race, body mass index, education, and current and lifetime smoking, the ORs for persons with dust and fume exposures for chronic cough, chronic phlegm, persistent wheeze, and GOLD stages 2 and higher COPD were significantly elevated and similar for men (1.83,1.84, 2.0, 1.61, respectively) and women (1.65, 1.82, 1.98, 1.90, respectively). Monsó^[15]^ conducted a study on 105 nonsmoking animal keepers working in enclosed buildings and the results showed that a high concentration of organic dust in enclosed buildings may be the main cause of COPD in these workers. Under conditions of exposure to high concentrations of organic dust, the OR (95% CI) value was 5.38 (1.17–24.74). After adjusting the covariates, temperature, humidity, area, and endotoxins in indoor pollutants in enclosed buildings, the OR (95% CI) value was 6.60 (1.10–39.54); which was still statistically significant. Vinnikov^[16]^ identified 458 factory workers with FEV_1_/FVC < 70%, FVC < the lower limit of normal, or FEV_1_ < the lower limit of normal based on the results of lung function measurements. After adjusting for confounding factors, such as age, gender, and dust exposure duration, the results showed that workers exposed to high concentrations of dust in the workplace were twice as likely to develop COPD compared to those exposed to low concentrations. Therefore, it can be inferred, that the higher the dust exposure concentrations, the more possibility of developing COPD. In this study, we analyzed the relationship between occupational risk factor exposure and COPD severity and found that the exposure level was related to the severity of COPD. When setting the low exposure level as a reference, medium and high exposure levels were significantly related to the severity of COPD, especially in the high exposure level group.

Möhner^[17]^ surveyed 1421 quartz stone processing workers and found that, on average, 1 mg/m^3^ of quartz dust exposure would reduce the FEV_1_/FVC by 2.75% (*P* < .001) and increase the risk of COPD by 1.81 times. In this study, we found that cumulative exposure was linearly negatively correlated with FEV_1_%. The higher the cumulative exposure to occupational risk factors, the worse the patient’s lung function was.

Work-related COPD and non-work-related COPD have the same pathogenesis. The length of the incubation period from exposure to occupational risk factors to the appearance of respiratory symptoms can vary based on the nature, concentration, and time of exposure to occupational hazard factors. Therefore, there was uncertainty in determining the severity of COPD by the length of service. For this reason, revision of the 5 years of occupational service requirement in the standard for diagnosing work-related COPD should be considered.

### 4.3. Special measures for work-related COPD prevention and treatment

The ebb and flow of the early symptoms of work-related COPD are closely related to exposure to occupational risk factors in the work environment.^[18]^ Therefore, early detection, timely separation, and intervention treatment are of great value for postponing the prognosis of work-related COPD. If COPD patients are exposed to occupational risk factors during work, they should be asked to leave the workplace as soon as possible, and to avoid contact with smoke, fog, dust, and other risk factors in the work environment. Patients in the acute exacerbation stage can be treated with anti-inflammatory agents supplemented with nutritional support to prevent complications; patients in the stable stage are given symptomatic and supportive treatment to relieve symptoms and improve lung function.^[19]^ Currently, there is no special treatment for work-related COPD, the treatment principles are the same as that for other work-related lung diseases. Exposure prevention is the priority measure to prevent occupational risk factors from affecting workers’ respiratory health.^[20]^ First, pre-employment occupational health examinations should be conducted for workers exposed to occupational risk factors to assess their respiratory health. Individuals with a history of asthma should be prohibited from engaging in work-related COPD occupations. At the same time, in accordance with the requirements of the Occupational Disease Prevention and Control Law, the protection of workers should be strengthened. For example, highly toxic materials should be replaced by nontoxic or low-toxic materials. And at the same time, the content of toxic substances in materials should be monitored regularly. Innovation in the operating environment process flow; automated, mechanized, and closed industrial ventilation production processes should be encouraged as much as possible. Regularly maintain mechanical equipment to prevent running, emissions, drips, and leaks. Pre-employment or regular training for workers who are exposed to occupational risk factors is all necessary. When FEV_1_ decreases from baseline by 10.0 to 15.0% within 1 year, information about the occupational history should be inquired in time to rule out occupational risk factor exposure. For workers exposed to occupational hazards whose lung function declines rapidly, further examinations and occupational health risk assessments need to be carried out through lung function tests or existing health monitoring programs. The National Institute of Occupational Safety and Health in the United States has developed a job exposure matrix (JEM) to evaluate COPD, which can assess the risk of COPD development through the level of exposure to occupational hazards.^[21,22]^ Doney^[23]^ showed that there was a clear correlation between COPD and JEM assessment of COPD by measuring lung function. General personal occupational health risk assessment results may be affected by various biases, such as recall bias, but JEM is a reliable monitoring method for reducing or eliminating personal recall bias based on the level of exposure to occupational risk factors (low, medium, or high-level exposure).

GOLD has been updated to version 2021, and has gradually improved the diagnosis, evaluation, and treatment of COPD.^[24]^ However, general hospital clinicians’ understanding of work-related COPD remains inadequate. In this study, 26.0% of the patients had a history of exposure to occupational risk factors, but no detailed occupational risk factor exposure documents related to this disease were found in the medical records. Therefore, it is necessary to increase the awareness of respiratory health clinicians to inquire the occupational exposure history during their clinical practice. In one study among 21 patients diagnosed with work-related COPD, 76% of whom were <60 years of age.^[4]^ They found that the incubation period of the disease in the exposure group was significantly shorter than that in the non-exposure group. Therefore, the age of onset of work-related COPD patients may be younger than ordinary COPD patients. Since there were no obvious specific symptoms and unique differential diagnosis indicators, it was generally difficult to distinguish between occupational and non-occupational pathogenic factors, such as smoking. Therefore, the identification of causal factors is critical in the diagnosis of work-related COPD. In the diagnosis of COPD caused by occupational risk factors, it is necessary to comprehensively investigate the patient’s occupational history, onset time, course of the disease, and occupational exposure dynamic relationship, especially for patients engaged in agriculture, forestry, animal husbandry and fishing, water conservation, mining, and manufacturing.

## 5. Conclusions

Among 4082 investigated COPD patients in this follow-up survey, 26% of cases had a history of exposure to occupational risk factors. The top 3 industries with a large COPD case number and a history of occupational risk factor exposure ranked in the order of agriculture (including farming, forestry, animal husbandry, and fishery), manufacturing, and mining. The incubation period of COPD in the exposure group was significantly shorter than that in the non-exposure group. The exposure level of occupational hazards was an important risk factor affecting the severity of COPD. In addition, a higher occupational risk factor exposure rate was found in the lower education level cases.

Although this retrospective investigation showed a significant relationship between occupational risk factor exposure and the severity and progression of COPD, a further cohort study is needed to verify this conclusion.

## Acknowledgments

We thank the financial support from the Key Research and Development Plan of Shandong Province [grant number 2019GSF111025] and the Chinese Center for Disease Control and Prevention Health standard-setting project [grant numbers20210303]. The excellent assistance of Dr Patel Sefali in the preparation of this article is greatly appreciated.

## Author contributions

**Formal analysis:** Yao Chen, Rui Yuan.

**Investigation:** Cong Peng, Yu Cai, Hua Zhang, Pingping Song.

**Supervision:** Yongjian Yan.

**Writing – original draft:** Yao Chen, Cong Peng.

**Writing – review & editing:** Chunling Zhang, Yongjian Yan.

## Supplementary Material


