# Supplementary material for: Exposure to occupational risk factors is associated with the severity and progression of chronic obstructive pulmonary disease
Source: Medicine (Baltimore). 2023 Feb 10;102(6):e32908. doi: 10.1097/MD.0000000000032908 (PMC9907959; doi:10.1097/MD.0000000000032908)
Supplement: Supplementary file 1 [file medi-102-e32908-s001.pdf]

Questionnaire on exposure to occupational risk factors for the onset and exacerbation of chronic obstructive pulmonary disease

Q1: Personal information:

Gender:____ Age:____

Age of onset:____ Education level:____

Smoking history: ☐Current smoker ☐Former smoker ☐Never smoker

Q2: Occupational exposure history

1. What type of industry do you work in specifically?
2. What occupational health hazards are you likely to be exposed to at work
   1) ☐No occupational hazard factors
   2) ☐Dust:
      A. ☐Inorganic dust (mineral dust, carbon-containing dust, metallic dust, artificial inorganic dust)
      B. ☐Organic dust (animal dust, plant dust, artificial organic dust)
      C. ☐Mixed dust
   3) ☐Irritating chemicals:
      A. ☐Acids and acid-forming compounds
      B. ☐Aldehydes
      C. ☐Nitrogen oxides
      D. ☐Metal compound smog
      E. ☐Organic solvents
      F. ☐Chlorine and its compounds
      G. ☐Ammonia
   4) ☐Fumes
   5) ☐Unclear
3. Exposure duration:____
4. Exposure level (value)
   1) ☐Low exposure(Low exposure level applied to workplaces with advanced technology, high degree of automation, mechanization, airtightness, clean working environment, and better protective measures.)
   2) ☐Medium exposure(Medium exposure level applied to workplaces with average technological advancement, average automation, mechanization, airtightness, environmental and personal protection, but the protection measures could not achieve the expected results.)
   3) ☐High exposure(High exposure level applied to workplaces with backward technology, low level of automation/mechanization, manual or non-enclosed operation environment, no environmental protection or ineffective protection,

and no personal protection or ineffective protection.)

5. FEV 1 /FVC:____ FEV_1%____
